# Restriction Spectrum Imaging Differentiates True Tumor Progression From Immune-Mediated Pseudoprogression: Case Report of a Patient With Glioblastoma

**DOI:** 10.3389/fonc.2020.00024

**Published:** 2020-01-28

**Authors:** Shadi Daghighi, Naeim Bahrami, William J. Tom, Nicholas Coley, Tyler M. Seibert, Jona A. Hattangadi-Gluth, David E. Piccioni, Anders M. Dale, Nikdokht Farid, Carrie R. McDonald

**Affiliations:** ^1^Center for Multimodal Imaging and Genetics, University of California, San Diego, La Jolla, CA, United States; ^2^Department of Radiology, University of California, San Diego, La Jolla, CA, United States; ^3^Department of Pathology, University of California, San Diego, La Jolla, CA, United States; ^4^Department of Radiation Medicine, University of California, San Diego, La Jolla, CA, United States; ^5^Department of Neurosciences, University of California, San Diego, La Jolla, CA, United States; ^6^Department of Psychiatry, University of California, San Diego, La Jolla, CA, United States

**Keywords:** brain tumors, restriction spectrum imaging, immunotherapy, pseudoprogression, diffusion imaging

## Abstract

Immunotherapy is increasingly used in the treatment of glioblastoma (GBM), with immune checkpoint therapy gaining in popularity given favorable outcomes achieved for other tumors. However, immune-mediated (IM)-pseudoprogression is common, remains poorly characterized, and renders conventional imaging of little utility when evaluating for treatment response. We present the case of a 64-year-old man with GBM who developed pathologically proven IM-pseudoprogression after initiation of a checkpoint inhibitor, and who subsequently developed true tumor progression at a distant location. Based on both qualitative and quantitative analysis, we demonstrate that an advanced diffusion-weighted imaging (DWI) technique called restriction spectrum imaging (RSI) can differentiate IM-pseudoprogression from true progression even when conventional imaging, including standard DWI/apparent diffusion coefficient (ADC), is not informative. These data complement existing literature supporting the ability of RSI to estimate tumor cellularity, which may help to resolve complex diagnostic challenges such as the identification of IM-pseudoprogression.

## Background

In patients with GBM, surgical resection, followed by radiotherapy (XRT) with concomitant and adjuvant temozolomide is the standard of care (1, 2). In up to 30% of patients, areas of new or increasing enhancement and/or edema are seen on standard MRI within the radiation field within the first few months following treatment (3). These findings mimic tumor progression; however, they often resolve or stabilize without intervention. This phenomenon is referred to as *pseudoprogression*. Although the underlying pathophysiology is unknown, it is thought to represent treatment effect (3). In fact, patients who develop pseudoprogression have overall improved survival rates (4). Due to the complexity of differentiating pseudoprogression from true progression, current guidelines established for evaluating response to therapy in high-grade glioma [i.e., Response Assessment in Neuro-oncology; RANO (5)] dictate that progressive disease should not be diagnosed on imaging within the first 3 months following completion of XRT unless there is: (1) new enhancement outside the main radiation field or; (2) pathologic confirmation of unequivocal tumor progression.

Recently, a new type of pseudoprogression has emerged, further confounding the evaluation of treatment response in patients with brain tumors. This type of pseudoprogression follows treatment with immunotherapy. Immunotherapy is a broad term which refers to several different types of therapeutic agents, that target tumor cells by harnessing the patient's own immune system. These agents include checkpoint inhibitors, vaccines, CAR-T therapy, and viral therapy (6). Following treatment with immunotherapy, an immune reaction occurs in the tumor bed with additional systemic effects throughout the brain (7). As a result, areas of enhancement and edema appear within and possibly distal to the primary tumor site. As with standard pseudoprogression, *immune-mediated* (IM)-pseudoprogression can mimic true tumor progression (8). However, IM-pseudoprogression may occur later than standard pseudoprogression and may occur distal to the primary site of tumor (9). This has led to the development of the immune RANO (iRANO) criteria (9). According to these criteria, progressive disease should not be diagnosed on imaging within the first 6 months following initiation of immunotherapy. Given the profound implications for patient care, the ability to differentiate true progression from IM-pseudoprogression is of critical importance in Neuro-Oncology. Misdiagnosis could potentially delay survival-prolonging treatments in patients with true progression, while resulting in unnecessary surgical, or medical interventions in patients with IM-pseudoprogression.

Conventional imaging alone has failed to differentiate IM-pseudoprogression and true tumor progression, as both are characterized by areas of increased contrast enhancement and edema (10). To date, very few studies have utilized advanced neuroimaging techniques to address the issue of IM-pseudoprogression despite its increased recognition (11–13). Among these, diffusion-weighted imaging (DWI) has generated attention due to its proposed ability to provide a surrogate marker of changes in tumor cellularity. However, it is well-appreciated that DWI-derived apparent diffusion coefficient (ADC) values are influenced by both edema and necrosis within and surrounding the tumor bed. Because the immune response may increase edema, lymphocytic infiltration, and necrosis, the effects of these pathologies on the ADC signal may reduce its ability to differentiate true progression from IM-pseudoprogression.

An advanced DWI method called *restriction spectrum imaging* (RSI) has emerged as a promising new technique for estimating tumor cellularity and has demonstrated initial efficacy for evaluating treatment response in patients with GBM and other high-grade gliomas (14). Unlike standard DWI, RSI utilizes multiple *b*-values (*b* = 0, 500, 1,500, and 4,000 s/mm^2^ with 1, 6, 6, and 15 unique gradient directions for each *b*-value, respectively) and diffusion times to separate different components of the diffusion signal, distinguishing the spherically-restricted diffusion signal associated with tumor cellularity from hindered diffusion signal that is influenced by edema and inflammation in the extracellular matrix (15–18). Because the immune response may increase edema, necrosis, and lymphocytic infiltration, we tested whether RSI could offer an advantage for differentiating true progression from IM-pseudoprogression.

We present a case of a patient with GBM who developed IM-pseudoprogression after initiation of a checkpoint inhibitor. This patient eventually developed true tumor progression at a separate location. While both the IM-pseudoprogression and the true tumor progression were characterized by regions of marked contrast enhancement and edema, the two could be readily distinguished using RSI, whereas differentiating these entities on ADC was quite challenging.

## Case Presentation

**Figure 1:**
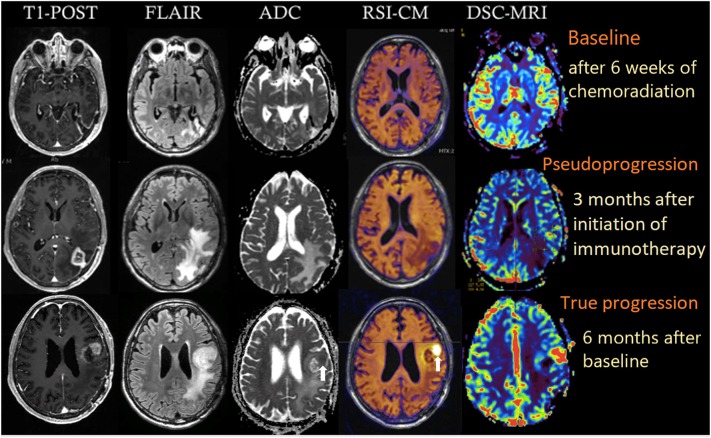
Multiparametric imaging of a patient with GBM at baseline (after 6 weeks of chemoradiation; top row), 3 months after initiation of nivolumab (middle row), and after another 3 months (bottom row). White arrows depict the regions of true tumor progression on ADC and RSI-CM. All images were acquired on a 3T GE MR750 system. RSI-CM, restriction spectrum imaging cellularity map.

**Figure 2:**
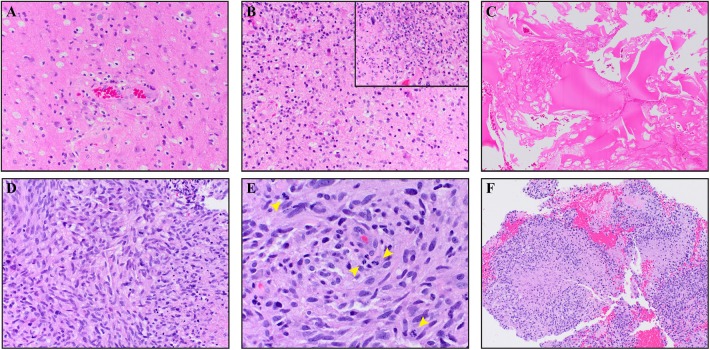
The top panels **(A–C)** are from the IM pseudoprogression time-point, demonstrating paucicellular, mitotically quiescent tissue whereas the bottom panels **(D–F)** are from the true progression time-point demonstrating glioblastoma. The IM pseudoprogression lesion contains abundant foamy macrophages **(A)** in addition to a mixed chronic inflammatory infiltrate **(B)**. Some regions also show neutrophils and fragments of neutrophilic debris (insert **B**). Foci of geographic coagulative necrosis, attributable to radiation effect, are also present **(C)**. The glioblastoma shows highly cellular, moderately pleomorphic, predominantly spindle-shaped tumor cells **(D)** with brisk mitotic activity (arrowheads **E**) and regions of serpiginous pseudopallisading necrosis **(F)**. Magnification of the images is as follow: (**C,F** × 100. **A,B,D** × 200. **E,B** × 400). All images are taken from hematoxylin and eosin stained, formalin-fixed paraffin-embedded tissue.

A 64-year-old male with no family history of cancer presented with blurry vision in his right visual field, headache, and difficulty with multitasking and writing. Initial MRI (3T GE MR750 system) demonstrated a large enhancing mass in the left temporal-occipital lobe. The patient underwent gross total resection (GTR) (Figure 1, top row), and pathology showed GBM, IDH-wild type (Ki-67 40%, MGMT unmethylated, EGFR amplified). Following surgery, he received 6 weeks of radiation (60 Gy/30 fractions) and concurrent temozolomide (TMZ; 140 mg/night). One month following completion of radiation, he began therapy with a checkpoint inhibitor (nivolumab 240 mg IVq 2 weeks). Three months following initiation of nivolumab, the patient developed an area of heterogeneous enhancement around the surgical cavity with new confluent surrounding FLAIR hyperintensity (Figure 1, middle row). Whereas, the ADC was equivocal, there was no elevated signal on RSI or increased relative cerebral blood volume (rCBV) on dynamic susceptibility contrast (DSC) perfusion MRI. The patient had no changes in his clinical symptoms. Despite this, due to suspicion of tumor progression, the patient underwent a second resection. Histopathology revealed a lesion with abundant foamy macrophages in addition to a mixed chronic inflammatory infiltrate, neutrophils, and fragments of neutrophilic debris. Foci of geographic coagulative necrosis, attributed to radiation effect were also observed (Figures 2A–C). However, no viable tumor cells were detected, supporting a diagnosis of IM-pseudoprogression. Three months later, following treatment with nivolumab, the patient became dysarthric and his concentration and handwriting worsened. MRI revealed a new distant region of enhancement and FLAIR hyperintensity in the left posterior frontal lobe (Figure 1, bottom row), this time associated with elevated signal on RSI and increased rCBV. Although ADC did demonstrate subtle low signal at the site of enhancement at the time of true progression, this signal is very similar to that of the normal appearing white matter, while the elevated RSI signal within this region is much more conspicuous (see Figure 1; white arrows). The region was biopsied, and pathology showed highly cellular, moderately pleomorphic tumor cells with brisk mitotic activity and regions of serpiginous pseudopallisading necrosis. These findings confirmed recurrent GBM (Figures 2D–F). The area of IM-pseudoprogression at the original surgery site had resolved in the interim. Thereafter, there were no further imaging changes at this site.

**Figure 3:**
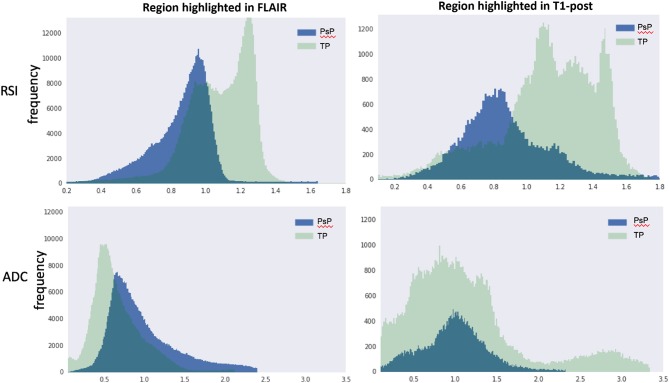
RSI (top row) and ADC (bottom row) histograms extracted from volumes manually defined by FLAIR hyperintensity (left column) and contrast enhancement (right column). The y-axis represents frequencies and the x-axis represents intensity values within each volume. PsP, pseudoprogression; TP, true progression; RSI, restriction spectrum imaging cellularity; ADC, apparent diffusion coefficient.

Quantification of the RSI and ADC signals within regions of contrast-enhancement and FLAIR hyperintensity were performed to further support our visual analysis (Figure 3). Inspection of RSI signal intensity histograms in Figure 3 (top row) reveals peak frequencies that are much higher for true progression relative to IM-pseudoprogression and mostly non-overlapping intensity distributions in volumes defined by both contrast enhancement and FLAIR hyperintensity. Conversely, there is considerable overlap in the peaks and distribution of values for ADC in both contrast-enhancing and FLAIR hyperintense regions (Figure 3; bottom row), resulting in poor differentiation of true progression and IM-pseudoprogression. This is especially salient within the contrast-enhancing region where the mean RSI signal is higher for true progression (mean intensity = 1.00) than for IM-pseudoprogression (mean intensity = 0.89), whereas the ADC values within the contrast-enhancing region are nearly identical for true progression (mean intensity = 0.98), and IM-pseudoprogression (mean intensity = 0.97).

## Discussion

RSI is an advanced DWI technique that has been shown to improve tumor conspicuity in patients with high-grade glioma (16) and provide a better biomarker of response to therapy in patients on antiangiogenic agents, relative to standard DWI (17, 18). Furthermore, our previous work has shown that RSI estimates of cellularity are directly related to histopathological tumor cellularity in preclinical models (15, 19). The case presented here demonstrates that RSI may also be a promising tool for differentiating IM-pseudoprogression from true progression, potentially by isolating the restricted diffusion associated with cellularity and removing the hindered signal associated with edema, lymphocytic infiltration, and necrosis. This property of RSI provides an advantage over standard ADC, which is often dominated by changes in hindered diffusion (16). In fact, in a series of 21 children treated with a peptide-based vaccine for diffuse pontine gliomas, changes in ADC only modestly differentiated true progression from IM-pseudoprogression (12). The authors acknowledged that suboptimal differentiation was likely due to the inability of ADC to distinguish viable tumor (low ADC) from necrosis and vasogenic edema (high ADC). Thus, when measuring changes in ADC, the lower ADC values of a viable tumor likely blend in with the higher ADC values found in areas of necrosis and edema (see Figure 1; bottom row). As a result, current ADC models do not sufficiently meet the challenge posed by IM-pseudoprogression.

The case presented here demonstrates that RSI is able to differentiate pathologically proven IM-pseudoprogression from true progression in the *same* patient. This is supported by both visual inspection and quantification of the RSI cellularity maps. Whereas, the visual analysis is critical for making individual diagnostic decisions, quantification of the RSI signal could be used to differentiate IM-pseudoprogression from true progression in clinical trials and/or when visual inspection is equivocal. In terms of feasibility, RSI has already been seamlessly integrated into the clinical workflow at our center as well as several others. The sequence takes ~4 min on most 3T MRI systems, is post-processed on a separate workstation within ~5 min of completion of the scan, and the post-processed RSI map is then sent back to PACS as the final series of the MRI scan. Furthermore, the clinical interpretation of the RSI map is analogous to that of DWI, albeit with greater conspicuity and specificity, making the output highly familiar to radiologists. It is important to note that in our case the RSI signal was concordant with results from DSC-MRI at both time-points. Thus, RSI-based measures of cellularity provide complementary information to rCBV, and therefore may be used in combination with perfusion imaging to increase confidence in diagnosing IM-pseudoprogression.

In addition to immunotherapy, our patient received standard chemoradiation with evidence of radiation effect on histopathology. Therefore, it is important to consider that the pseudoprogression observed in our patient may reflect a combination of standard chemoradiation and immunotherapy. However, the presence of inflammatory cells on histopathology and the timing of the MRI changes (>3 months following chemoradiation) increases the likelihood of IM-pseudoprogression. This case demonstrates that RSI could be used to prevent unnecessary biopsy or surgical interventions in the case of pseudoprogression in patients treated with immunotherapy.

## Data Availability Statement

The datasets generated for this study are available on request to the corresponding author.

## Ethics Statement

The studies involving human participants were reviewed and approved by Human Research Protection Program at the University of California, San Diego. The patients/participants provided their written informed consent to participate in this study. Written informed consent was obtained from the individual(s) for the publication of any potentially identifiable images or data included in this article.

## Author Contributions

SD, NF, and CM wrote the case report. NB, DP, NC, JH-G, WT, AD, and TS reviewed and edited the manuscript.

### Conflict of Interest

AD is the developer of the RSI sequence (patent pending). The remaining authors declare that the research was conducted in the absence of any commercial or financial relationships that could be construed as a potential conflict of interest.
